# Hospital Ships and the Right of Search: The Practical Lesson of the *Ophelia* Case

**Published:** 1915-07-03

**Authors:** 


### HOSPITAL SHIPS AND THE RIGHT OF SEARCH.

### The Practical Lesson of the "Ophelia" Case.

SOME time ago THE HOSPITAL contained an article describing and defining the position in international law of hospital ships. It was there explained how by Convention 10 of the Hague Conference, 1907, the principles of the old Geneva Convention of 1864 were adapted to the circumstances of modern warfare, and the provisions of the Hague Convention as to the various classes of hospital ships were there detailed. In the case of the *Ophelia*, recently before the Prize Court, occasion has arisen for the practical consideration of these rules. She was a steamship captured in October 1914, and the Crown claimed that she should be condemned as lawful prize, but a claim for her release was made by the Government of the German Empire on the ground that she was a military hospital ship. Both Governments based their contentions on the Hague Conventions, and in delivering the judgment of the Court the President said: "I will deal with the case upon the same basis, avoiding all inquiry whether the Government of the German Empire is or is not precluded by its conduct of the present war from invoking the aid or protection of the tenth or any other of the Hague Conventions."

### THE LAW AND THE DECISIVE POINT.

The law as laid down in Convention 10 is very plain. If a hospital ship complies with it she is entitled to protection, but if she is used for any military purpose, either for aiding her belligerent owner militarily or for injuring the enemy, her protection is lost. That the staff is armed, for the purpose of maintaining order or for defending the wounded or sick, and that an apparatus for wireless telegraphy is on board, are not sufficient reasons for withdrawing the protection. The whole question, therefore, was whether the *Ophelia* was used for military purposes.

In endeavouring to arrive at an answer to that question, the Court reviewed at great length the evidence, oral and documentary, put before it, the ship's log and her movements were considered, her officers were examined, and her equipment and the use she made of it taken into account. The result may be given in the President's words: "The conclusions to which the evidence has compelled me to come are that the *Ophelia* was not constructed, adapted, or used for the special and sole purpose of affording aid and relief to the wounded, sick and shipwrecked, and that she was adapted and used as a signalling ship for military purposes. She has therefore forfeited the protection claimed under the Convention, and the decree of the Court is that the *Ophelia* being an enemy ship is condemned as prize."

While the case thus depended on its own special circumstances, the President in the course of his judgment touched on several points of general importance as interpreting and applying the bare statements in the Convention.

### POINTS IN THE JUDGMENT.

These principal points of interest may be summarised thus:

First, the movements of the *Ophelia* were suspicious leading to the inference that she wished to avoid British war vessels, whereas "those in command of a genuine hospital ship on a humane quest would not fear any harm or ill-treatment from any war vessel"; second, the equipment and lighting and signalling apparatus of the vessel were not wholly designed for hospital purposes; and third, after the ship was captured and brought to the Thames the staff surgeon in charge of her destroyed certain of the ship's documents. That was a damning circumstance, for the Prize Courts have always very seriously regarded the "spoliation of documents" by any ship, and the principle was held to apply equally forcibly, to say the least, to documents which would throw light upon the way in which a ship purporting to be a hospital ship had been employed. On this last point the observations of the President are strong and explicit—"About the innocence of hospital ships from engaging in warlike services there ought to be no question. Their records should be clean. If they are so, their preservation would be an additional safeguard against capture. If they are not preserved but destroyed, the inference that if produced they would be silent but eloquent witnesses of guilty practices would be strong. As to some of the documents on board, such as the secret codes for wireless telegraphy, the law officers of the Crown did not complain of their destruction. In my view, even documents like these ought not to be destroyed. They might be required in order to test the accuracy of the versions given of messages sent and received. But whatever might be said in justification or palliation of the destruction of documents of this nature by reason of the orders of those in high command or otherwise, some of the other documents which were destroyed should certainly have been preserved or given up. Books recording messages transmitted or received by wireless telegraphy or by any form of signals directing the operations of the ship ought to be kept. If such messages related to the legitimate work of hospital ships they would not harm those in charge or prejudicially affect the ship itself. If they are destroyed on the eve of capture, no one could reasonably complain if unfavourable inferences were drawn. For the burning in November (some fortnight or more after the ship was captured) of the records of the various signalling lights which had been supplied, and which had been used on the ship since she set out as a hospital ship, I see no justification whatsoever. By the express terms of the Convention, the right of search of hospital ships is given to belligerents. If those in charge of such ships can with impunity destroy all the documents and records of the ship immediately before a searching officer boards her, the right of search becomes to a great extent nugatory."

